# Heterosis Derived From Nonadditive Effects of the *BnFLC* Homologs Coordinates Early Flowering and High Yield in Rapeseed (*Brassica napus* L.)

**DOI:** 10.3389/fpls.2021.798371

**Published:** 2022-02-15

**Authors:** Caochuang Fang, Zhaoyang Wang, Pengfei Wang, Yixian Song, Ali Ahmad, Faming Dong, Dengfeng Hong, Guangsheng Yang

**Affiliations:** ^1^National Key Laboratory of Crop Genetic Improvement, Huazhong Agricultural University, Wuhan, China; ^2^Hubei Hongshan Laboratory, Wuhan, China

**Keywords:** heterosis, flowering time, *BnFLC*, non-additive effects, additive effects, rapeseed, plant yield

## Abstract

Early flowering facilitates crops to adapt multiple cropping systems or growing regions with a short frost-free season; however, it usually brings an obvious yield loss. In this study, we identified that the three genes, namely, *BnFLC.A2*, *BnFLC.C2*, and *BnFLC.A3b*, are the major determinants for the flowering time (FT) variation of two elite rapeseed (*Brassica napus* L.) accessions, i.e., 616A and R11. The early-flowering alleles (i.e., *Bnflc.a2* and *Bnflc.c2*) and late-flowering allele (i.e., *BnFLC.A3b*) from R11 were introgressed into the recipient parent 616A through a breeding strategy of marker-assisted backcross, giving rise to eight homozygous near-isogenic lines (NILs) associated with these three loci and 19 NIL hybrids produced by the mutual crossing of these NILs. Phenotypic investigations showed that NILs displayed significant variations in both FT and plant yield (PY). Notably, genetic analysis indicated that *BnFLC.A2*, *BnFLC.C2*, and *BnFLC.A3b* have additive effects of 1.446, 1.365, and 1.361 g on PY, respectively, while their dominant effects reached 3.504, 2.991, and 3.284 g, respectively, indicating that the yield loss caused by early flowering can be successfully compensated by exploring the heterosis of FT genes in the hybrid NILs. Moreover, we further validated that the heterosis of FT genes in PY was also effective in non-NIL hybrids. The results demonstrate that the exploration of the potential heterosis underlying the FT genes can coordinate early flowering (maturation) and high yield in rapeseed (*B. napus* L.), providing an effective strategy for early flowering breeding in crops.

## Introduction

Rapeseed (*Brassica napus* L.) is one of the most important oil crops worldwide and comprises more than 55% of the total oilseed production in China. But with the change of cultivation systems for next-stubble crops (e.g., rice and corn) in central China, the growth season of rapeseed is being restricted. And in northwestern China, undetermined early frost is often a threat to seed maturation. Thus, a shorter growing season or earlier maturation time would enable increased cultivation area and production for rapeseed in China. As flowering time (FT) is often associated with maturation time in crops (Wang Y. et al., 2021), the implementation of shorter growth stages *via* regulating FT becomes a feasible idea.

Advantageously, extensive genetic research on FT genes and qualitative trait loci (QTLs) has been conducted in rapeseed. In its close relative model plant *Arabidopsis*, nearly 306 genes have been reported in the FT regulatory network which are involved in vernalization pathway, autonomous pathway, photoperiod pathway, ambient temperature pathway, and gibberellins pathway (Bouché et al., 2016). This provides a reliable reference for FT in rapeseed (Schiessl et al., 2014; Bouché et al., 2016). In rapeseed, many QTLs and peaks identified *via* genome-wide association studies have been colocalized with some of the annotated FT genes from the reference genome (Schiessl, 2020), and the crucial genes, namely, *BnFLC.A10*, *BnFLC.A2*, and *BnFLC.C2*, which participate in the vernalization pathway, have been characterized in rapeseed (Chen L. et al., 2018; Yin et al., 2020).

However, many studies show that FT genes often induce pleiotropic effects in multiple traits including yield in some crops. In rice, *OsWOX13* not only triggers early initiation but also enhances drought tolerance (Minh-Thu et al., 2018). Mutations of *Osgi* and *Ospho2* delay initiation and reduce biomass but promote Pi concentration in rice (Li et al., 2017). The NAC transcription factor gene *OsY37* accelerates heading and also promotes leaf senescence in rice (Mannai et al., 2017). *OsWDR5a* interacts with *OsTrx1* to promote heading, secondary branches, and grain production (Jiang et al., 2018). *SDG701* promotes heading in rice, but some spikelets failed to produce seeds in heterozygous *sdg701-1 (+/–)* mutant plants, which decreased grain yield (Liu et al., 2017). *OsDHD1* interacting with *OsHAP5C/D* not only delays heading but also enhances yield (Zhang et al., 2019). *OsMFT1* delays heading in rice by suppressing *Ehd1*-, *FZP*-, and *SEPALLATA-*like genes, and increases the number of spikelets per panicle (Song et al., 2018). The *RFT 1* allele from rice cultivar Zhenshan 97 delayed heading and also increased plant height (PH), grain weight, number, and yield compared to Milyang 46 (Zhu et al., 2017). In soybean, the mutation *Glyma.04G050200.1*, which is an ortholog of the *Arabidopsis thaliana EARLY FLOWERING 3* gene, delayed flowering and also increased grain weight per plant under short-day conditions (Lu et al., 2017). In rapeseed, an association panel comprised of 195 inbred lines revealed that allelic variations of FT genes *FD* and *FES1* were associated with PH (Kaur et al., 2021). In the double haploid (DH) population derived from Skipton/Ag-Spectrum/Skipton rapeseed, FT was negatively correlated with yield but positively correlated with PH under long-day photoperiodic conditions (Raman et al., 2019). It is obvious that early FT alleles usually induced yield loss, and late-flowering alleles induced yield gain.

Interestingly, some studies showed that FT genes had a strong positive effect on the heterosis of yield traits. In tomatoes, *SFT* homologs to *AtFT* integrated the flowering signal and facilitated flowering. The hybrid between *SFT* and its mutant *sft* showed a strong heterosis which was originated from overdominance that increased yield by up to 60% (Krieger et al., 2010). In rapeseed, *BnFT* and *BnTFL1* paralogs not only promote flowering but also modulate yield-related productivity traits (Guo et al., 2014). Indeed, F_1_ hybrids between *BnC6FTb*_*G*2154*A*_ missense mutants and controls generated higher seed yields (Guo et al., 2014). Mutations of the *BnTFL1-2* paralog had no significant effects on FT but affected yield components. Indeed, F_1_ hybrids between *BnTFL1-2* mutants and non-mutated parents had increased seed number per silique (SSN) and total seeds per plant, suggesting that heterozygous mutations in a *TFL1* paralog may impact heterosis in rapeseed (Guo et al., 2014).

Therefore, to adopt new cropping systems and short-season growing regions by rationally designing FT, the pleiotropic effect associated with FT genes, especially yield loss, should be carefully considered. Here, we dissected the major genetic components controlling the basis of FT variations between two elite inbreeding lines, i.e., 616A and R11, and produced a series of homozygous, as well as hybrids, to evaluate the genetic effects of the *FLC* homologs on FT and plant yield (PY). The results demonstrate that the exploration of heterosis from FT genes can coordinate early FT (early maturation) and high yield in rapeseed, providing an effective and indicative strategy for early maturation breeding in rapeseed and other crops.

## Materials and Methods

### Plant Materials

Four *B. napus* inbred accessions were used in this study. 616A, the maternal line of several elite rapeseed hybrids in China, is an elite temperature-sensitive polima cytoplasmic male sterile (pol CMS) accession, which shows early flowering in both winter and spring environments. 616A is a DH line from the F_1_ cross between a pol CMS line 987A (the female parent of a commercialized cultivar Heshengyou 868) and a pol CMS restorer 7,492 (Wang Z. et al., 2021). R11 acts as a semi-winter pol CMS restorer (Chen L. et al., 2018), which is an early-flowering mutation from the restorer R15 (the male parent of a commercialized hybrid cultivar Shengguang 101). ZS4R and 621R are also two semi-winter pol CMS restorers of several commercial hybrids released in China. A DH population with 352 independent genotypes is constructed from the F_1_ between 616A and R11 by microspore culture. All materials were bred by Huazhong Agricultural University.

### Field Experiment and Trait Evaluation

#### Field Experiments

The 352 DH lines and the two parents were randomly grown in two winter environments (i.e., Jiangling and Wuhan, Hubei) for two consecutive years, i.e., 2014–2015 and 2015–2016 seasons, and in one spring environment (Minle, Gansu) in the 2015 season. Two rows were set as a plot for each of the DH line, with 10 plants in each row.

The eight near-isogenic lines (NILs) (shown in the sections “Construction of Eight NILs for the FT Genes *BnFLC.A2*, *BnFLC.C2*, and *BnFLC.A3b*” and “The Development of Eight NILs Differs at the Loci of *BnFLC.A2*, *BnFLC.C2*, and *BnFLC.A3b*”), 19 NIL hybrids (shown in the section “Heterozygous Status in *BnFLC.A2*, *BnFLC.C2*, and *BnFLC.A3b* Generated Accumulative Heterosis in Plant Yield via Nonadditive “Effects”), and 16 non-NIL hybrids (shown in the section “Heterosis in Plant Yield Is Also Associated With Heterozygous *BnFLC.A2*, *BnFLC.C2*, and/or *BnFLC.A3b* Genotypes in Non-NIL Hybrids”) were planted as randomized blocks during the 2019–2020 season in two winter environments and one spring environment, with five plot repeats for the eight NILs and four plot repeats for the 19 NIL hybrids, 16 non-NIL hybrids, and two pol CMS restorers (i.e., ZS4R and 621R) in each of the three environments.

In all the field experiments, the row and plant spacing were 27 and 18 cm, respectively.

#### Phenotyping of Agronomic Traits of the Plots

FT was recorded as the number of days to flowering (DTF, in days) from sowing to the day when 50% of plants in the plot flowered. Eight plants for each plot of breeding tests in Jiangling and ten plants for each plot of breeding tests in Wuhan and Minle, excluding border rows, were randomly sampled to phenotype the following traits: branch number (BN), branch height (BH, distance from the cotyledonary node to the lowest branch in cm), plant height (PH, in cm), stem height (SH, distance from the cotyledonary node to the uppermost branch in cm), main raceme length (ML, in cm), silique number on main raceme (MSN), silique number per plant (PSN), PY (in g), silique length (SL, in mm), SSN, thousand seed weight (TSW, in g), oil content (OC, in %), protein content (PC, in %), and glucosinolate content (GC, in μmol/g).

### DNA and RNA Preparation, Molecular Markers, and QTL Mapping

The DNA was extracted from leaves according to a modified CTAB method (Allen et al., 2006). RNA was extracted from the first leaf from the top of 616A and R11 in the 4-week-old plants cultivated in Wuhan using the Eastep™ Super Total RNA Extraction Kit (Promega, Madison, WI, United States). cDNA was synthesized from RNA (4 μg samples) using the GoScript™ Reverse Transcription Mix (Promega) and then analyzed using the GoTaq™ qPCR Master Mix (Promega) and Bio-Rad CFX96 Real-Time System (Bio-Rad). The PCR program used was: 95°C for 1 min, and 40 cycles of 95°C for 10 s and 60°C for 30 s. The relative expression levels were calculated using the 2^–ΔΔCt^ method based on three biological samples and three replicates for each sample (Livak and Schmittgen, 2001). The expression levels of *BnFLC.A3b* were monitored by the specific primer, as shown in Supplementary Table 1 (Zou et al., 2012). The β-actin was used as the internal control (Wang et al., 2011).

The SCAR markers, i.e., STA2-55L/1R and STC2-4L/4R, were specifically amplified the wild-type (WT) alleles of *BnFLC.A2* and *BnFLC.C2*, respectively (Chen L. et al., 2018), while SCAR-5860/6430 was specifically amplified the nonfunctional allele of *Bnflc.a2* and *Bnflc.c2* in R11. *OPSNP7* was an intra-genetic marker specific for pol CMS restore gene *Rfp* (Liu et al., 2012). Insertion/deletion (InDel) markers for the candidate chromosomes were developed based on Illumina sequencing data of 616A and R11 referenced by the ZS11 genome (Supplementary Table 1; McKenna et al., 2010; Langmead and Salzberg, 2012; Song et al., 2020a). The 400 polymorphic SSR markers used for bulked segregant analysis (BSA) were obtained from a publication by Li et al. (2013). The sequence of the markers is shown in Supplementary Table 1.

The *BnFLC.A2*/*BnFLC.C2* and *Bnflc.a2*/*Bnflc.c2* subpopulations in the DH population from 616A and R11 were analyzed separately using the BSA strategy. For each of the subpopulation, the DNA of the 12 extremely early flowering and late-flowering individuals was used to build the bulk-DNA-pools, respectively. After the analysis by the 400 SSR markers, the co-segregated markers in the bulk-DNA pool, together with the InDel markers on chromosome A03, were used in the ICIM model and analyzed using QTL IciMapping software (Meng et al., 2015). The InDel markers on the candidate chromosomes were used to analyze the self-mapping population.

### Construction of Eight NILs for the FT Genes *BnFLC.A2, BnFLC.C2*, and *BnFLC.A3b*

The marker SCAR-5860/6430 described in the section “DNA and RNA preparation, molecular markers, and QTL mapping” for the genetic analysis of 616A and R11 was used to trace *Bnflc.a2* and *Bnflc.c2*. The candidate gene was traced using the co-dominant InDel marker in the linkage map and tested using the SCAR marker developed according to the sequence variation of the candidate gene between 616A and R11. Otherwise, *OPSNP7* was used to trace *Rfp* and distinguish restore lines and sterile plants (Liu et al., 2012). The polymorphism SSR markers randomly selected from the BSA analysis in the section “DNA and RNA preparation, molecular markers, and QTL mapping” were used for background selection. The NILs of both fertile and sterile plants were self-pollinated continuously for at least two seasons, producing eight near-isogenic restorer lines and eight male sterile lines.

The MGISEQ-2000-high-throughput sequencing platform (PE150; MGI Tech Co. Ltd., Wuhan, China) was used to perform genome sequencing of the eight near-isogenic restore lines (Korostin et al., 2020). After the raw data were filtered by fastp (version 0.20.0) (Chen S. et al., 2018), the clean data were aligned to ZS11 reference genome to get the sequence alignment/map (SAM) format file by Bowtie 2 (version 2.4.1) (Langmead and Salzberg, 2012; Song et al., 2020a) and converted into binary alignment/map (BAM) format file using Samtools (version 1.9) according to the mapping coordinates (Li et al., 2009). The Picard tools (version 2.23.2) were used to remove the PCR duplicates and calculate the genome coverage^1^. By means of the Genome Analysis Toolkit (version 3.8), the single nucleotide polymorphisms (SNPs) that were not only homozygous in 616A and R11 but also heterozygous between 616A and R11 were determined as effective (McKenna et al., 2010). In Khan et al. (2018), the recurrent parent genome recovery analysis of the eight NILs, the SNP homozygous in 616A was defined as 0, the SNP homozygous in R11 was defined as 2, and the heterozygous SNP was defined as 1. A genomic region covered by 15 continuous SNPs with 1 SNP step length, set as a sliding window (Huang et al., 2009), was defined as a recovery region when they were continuously scored less than eight (the threshold of the breakpoint). In Khan et al. (2018), the recurrent parent genome recovery is defined by the length of the recovery region dividing the reference genome length of ZS11.

### Statistical Analysis

The best linear unbiased estimation (BLUE) value was calculated using the restricted maximum likelihood (REML) linear mixed model in GenStat software. The genotypes of plant materials were set as fixed effects, whereas environment, plot, and randomly sampled plants were set as random effects.

The Pearson correlation coefficients were calculated by the BLUE values of the phenotypic data of the near-isogenic restorer lines and their hybrids using the package *PerformanceAnalytics* in R software (Dunne et al., 2018; Peterson and Carl, 2020).

Mid-parent heterosis was calculated *via* the BLUE values of hybrids and parental lines using the following formula: $\frac{H-\frac{1}{2}\left(p_{1}+p_{2}\right)}{\frac{1}{2}\left(p_{1}+p_{2}\right)}\cdot 100\%$ (Miedaner et al., 2016).

The genetic effects of the genes (QTLs) were calculated using the additive-dominant-epistasis model *via* the BLUE values of the phenotypic data of the NILs and their hybrids.

Multiple comparisons were conducted by least significance difference (LSD) using the base function in R.

## Results

### Allelic Variations in Multiple *FLC* Paralogues Are Associated With FT Difference Between 616A and R11

Both R11 and 616A showed an early flowering phenotype, with the FT of 140–143 and 136–138 days in the winter environment, and 57–61 and 58–60 days in the spring environment, respectively. Though R11 harbors the mutated alleles (i.e., *Bnflc.a2* and *Bnflc.c2*) at both *BnFLC.A2* and *BnFLC.C2* loci due to the segmentally homeologous exchange (HE) between chromosomes A02 and C02 (Chen L. et al., 2018), it showed significantly later flowering than 616A in the winter environment and no significant difference of FT with 616A in the spring environment. At the same time, what is the genetic reason that 616A has the early flowering phenotype in both the winter and spring environments?

**FIGURE 1:**
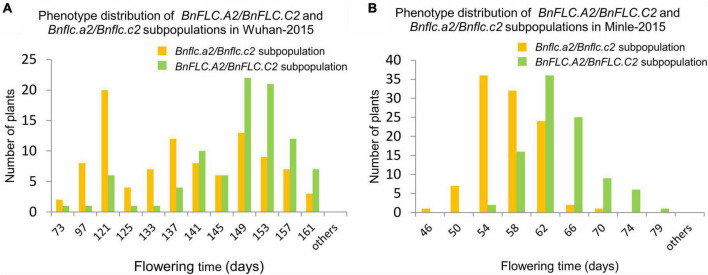
Histogram of phenotype distribution of the subpopulations from the double haploid population constructed by crossing 616A and R11 in the Wuhan-2015 and Minle-2015 environments. **(A)** Phenotype distribution of *BnFLC.A2*/*BnFLC.C2* and *Bnflc.a2*/*Bnflc.c2* subpopulations in Wuhan-2015. **(B)** Phenotype distribution of *BnFLC.A2*/*BnFLC.C2* and *Bnflc.a2*/*Bnflc.c2* subpopulations in Minle-2015. The yellow color represented the *Bnflc.a2*/*Bnflc.c2* subpopulation, and the green color represented the *BnFLC.A2*/*BnFLC.C2* subpopulation.

To understand the QTL (genes) conferring DTF variation between the parental accessions, we first analyzed the genotypes of *BnFLC.A2* and *BnFLC.C2* in 616A by STA2-55L/1R and STC2-4L/4R (Supplementary Table 1). The results showed that 616A carries the conserved WT alleles in both genes as reported before (Chen L. et al., 2018), which extremely significantly delayed the flowering in the DH population derived from the F_1_ cross between 616A and R11 (Supplementary Figures 1, 2). In addition, phenotypic variations in DTF remained wide in both of the WT “++/++” (*BnFLC.A2*/*BnFLC.C2*) and mutated “−−/−−” (*Bnflc.a2*/*Bnflc.a2*) subpopulations of the DH population (Figure 1 and Supplementary Figure 2). These observations indicated that although typical sequence variations in *BnFLC.A2* and *BnFLC.C2* between 616A and R11 mediate a significant FT alteration, there must be some other genes controlling early flowering in 616A.

**FIGURE 2:**
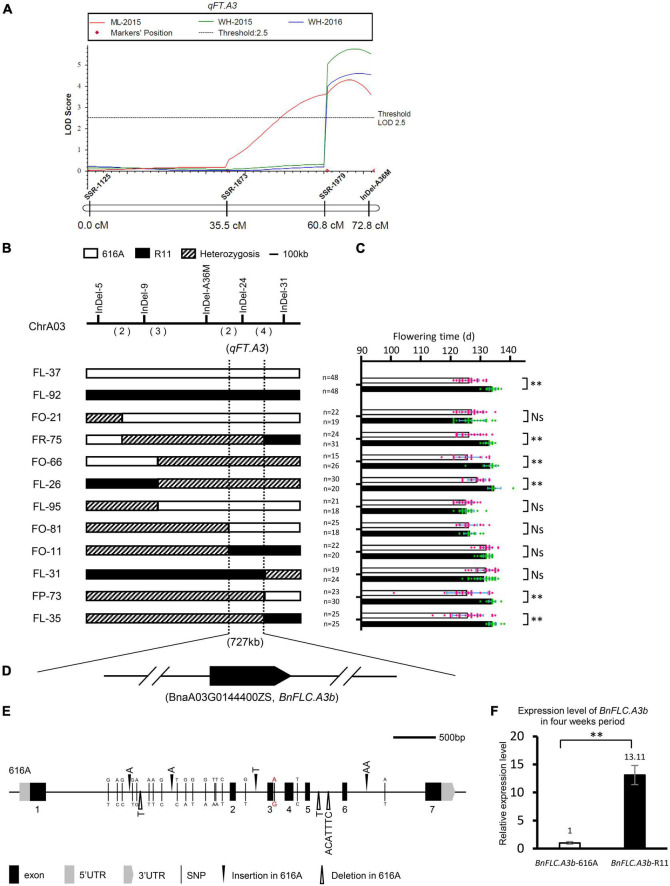
Qualitative trait locus (QTL) mapping of *qFT.A3*. **(A)** Partial linkage map of chromosome A03 and QTL scanning of *qFT.A3*. **(B)** Genotype of the recombinants in the BC_2_F_3_ population. The numbers in parenthesis indicate the number of crossovers. **(C)** Progeny testing of two near-isogenic lines (NILs) and 10 recombinants narrowed *qFT.A3* to a region of 727 kb. Hollow bars indicate the phenotype of 616A homozygous segments and solid bars indicate the phenotype of R11 homozygous segments. Data represent mean ± SD. NS, not significant; ^**^, significant at *p* < 0.01. **(D)** Annotated flowering time genes identified in the *qFT.A3* locus. Arrow indicated the direction of transcription; the break line indicated other genes not annotated for flowering time. **(E)** Sequence variations of the coding region of *BnFLC.A3b* in 616A and R11. The single nucleotide polymorphism and insertion/deletion variations between R11 and 616A were indicated by the benchmark of 616A. The red color indicated the G to A mutation of the five prime splicing sites between R11 and 616A. **(F)** Expression levels of *BnFLC.A3b* in 616A and R11, three biological replicates, and three technical replicates.

To explore the potential QTL (genes) rather than *BnFLC.A2* and *BnFLC.C2*, we constructed the extremely early and the late DNA bulks from the two subpopulations, respectively, and screened 400 polymorphic public SSR markers *via* the BSA strategy. In the two bulks, three co-segregated SSR markers, namely, SSR-1125, SSR-1873, and SSR-1979, were identified on chromosome A03 (Supplementary Figure 1 and Figure 2A). Then, an InDel marker, InDel-A36M, was developed from this chromosome. By using these four markers, we constructed a local genetic map and identified an FT QTL named *qFT.A3* from the genomic region delimited by the markers, which explained 7.6297, 6.7333, and 5.2631% of the phenotypic variation in FT of the DH population in the Wuhan-2015, Wuhan-2016, and Minle-2015 environments, respectively (Figure 2A). The early FT allele was inherited from 616A.

To narrow down the candidate interval of *qFT.A3*, more InDel markers were developed (Supplementary Table 1). Ten recombinant individuals were identified from the BC_2_F_3_ population between the InDel markers InDel-5 and InDel-31. The FT phenotypes of the corresponding BC_2_F_4_ progenies were investigated in the winter environment of Wuhan-2020. Based on this information, *qFT.A3* was further mapped into a 727-kb interval flanked by InDel-24 (Figures 2B,C). Genome annotation indicated that only one FT-related gene, *BnFLC.A3b*, resides in this candidate interval (Figure 2D and Supplementary Table 2; Song et al., 2020a).

Accordingly, we further amplified and comparatively analyzed the 1,691 bp promoter region and the coding region of *BnFLC.A3b* between the parental lines. Despite the conservation sequences in the seven annotated exons, seven InDels and 19 SNPs appeared in the introns, and five SNPs appeared in the promoter (Figure 2E). Among them, a G to A mutation in the five prime splicing site of the 3rd intron was very similar to the alternative splicing site of *FLC* orthologs, i.e., *BrFLC1* (6th intron G to A) and *BrFLC5* (3rd intron G to A), whose reduced expression levels were associated with the early FT of *Brassica rape* (Yuan et al., 2009; Xi et al., 2018). Consistently, we observed that the transcriptional level of *BnFLC.A3b* in R11 was nearly 13 times higher than that in 616A, suggesting the *BnFLC.A3b* allele in R11 was with a much stronger function than the allele in 616A (Figure 2F). In addition, similar sequence difference and expressing variations of *BnFLC.A3b* were also reported between Ningyou7 and Tapidor (Zou et al., 2012). Based on these results, we speculated that besides *BnFLC.A2* and *BnFLC.C2*, *BnFLC.A3b* is most likely to be the third gene leading to the FT variation between 616A and R11. At last, a sequence-characterized amplified region (SCAR) marker named SCAR-A37M was subsequently developed according to the seven-base pairs InDel to specifically amplify the allele of *BnFLC.A3b*-R11 for the next genotyping of breeding materials (Supplementary Table 1).

### The Development of Eight NILs Differs at the Loci of *BnFLC.A2*, *BnFLC.C2*, and *BnFLC.A3b*

**FIGURE 3:**
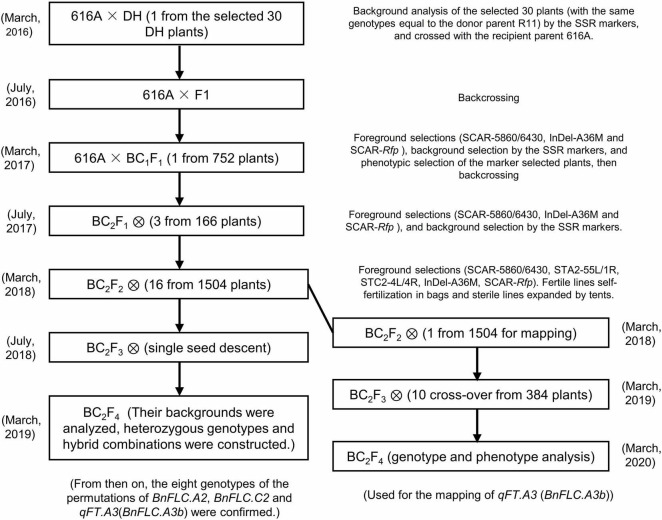
Selection scheme for the construction of the eight genotypes.

According to the abovementioned analysis, it is clearly suggested that the allelic variations of *BnFLC.A2*, *BnFLC.C2*, and *BnFLC.A3b* at least partially determined the FT difference between 616A and R11. To carefully evaluate the sole and conjunctive effects of these genes, we attempted to develop NILs with the different allelic permutations in the 616A background by marker-assisted foreground and background selections (Figure 3). Considering that 616A is a pol CMS line, the nuclear restorer gene *Rfp* for pol-CMS was simultaneously introduced from R11 during backcross to facilitate the trait investigation in the later studies.

**FIGURE 4:**
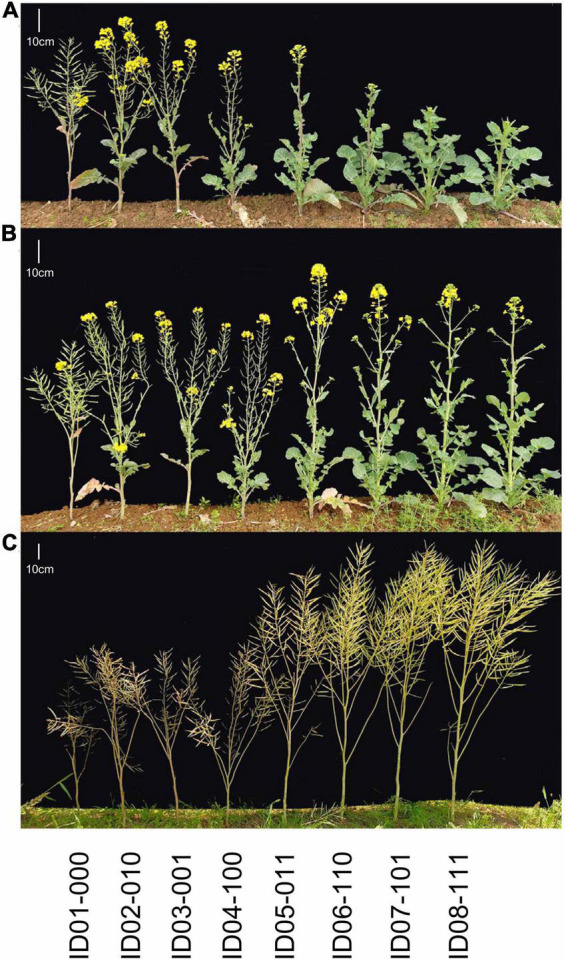
Continuous observation of growth status for the eight genotypes in the Wuhan environment. **(A)** February 21, 2020. **(B)** February 29, 2020. **(C)** April 28, 2020. From left to right: ID01-000, ID02-010, ID03-001, ID04-100, ID05-011, ID06-110, ID07-101, and ID08-111.

To reduce the breeding time, a DH line from the DH population coded as DH-8257, which shared the closest genetic background to 616A among the 30 DH lines with the same genotype as R11 at the three *FLC* target loci and *Rfp*, was chosen to backcross with 616A. After two rounds of foreground and background selection in BC_1_F_1_ and BC_2_F_1_ generations, the eight homozygous NILs of 616A and 616R (carrying the dominant *Rfp* allele) with permutations of *BnFLC.A2*, *BnFLC.C2*, and *qFT.A3* (*BnFLC.A3b*) were obtained in the resulting BC_2_F_2_ generation, respectively (Figure 3 and Table 1). Then, a single seed descent (SSD) method was adopted to progress the generation to BC_2_F_4_. The analysis of the genome resequencing data showed that the genome recovery of these eight fertile NILs varied from 92.72 to 98.0% (Table 1). The eight fertile NILs with the 616R background were defined as the eight genotypes, which were numbered from ID01-000 to ID08-111, in which “0” and “1” individually represent the existence of the homozygous mutation and WT genotypes of any of the three *BnFLC* loci (Figure 4 and Table 1).

**TABLE 1:** Allele permutations, recurrent genome recoveries (Khan et al., 2018), and flowering time comparison of the eight genotypes.

Genotypes ID	Allele permutations			Genome coverage	Recovery rate	Multiple comparisons of flowering time		
				
	*BnFLC.A2*	*BnFLC.C2*	*qFT.A3 (BnFLC.A3b)*			Jiangling	Wuhan	Minle
ID01-000	0	0	0	79.90%	94.62%	65.6 a	58.0 a	50.2 a
ID02-010	0	1	0	78.60%	97.52%	111.4 b	89.8 b	53.4 b
ID03-001	0	0	1	80.68%	92.72%	113.0 b	90.4 b	54.2 b
ID04-100	1	0	0	79.76%	95.97%	123.8 c	102.2 c	59.4 c
ID05-011	0	1	1	78.92%	95.36%	133.0 d	128.6 d	59.8 c
ID06-110	1	1	0	77.84%	98.0%	140.8 e	137.2 e	63.8 d
ID07-101	1	0	1	74.14%	96.47%	143.6 e	138.4 e	66.6 e
ID08-111	1	1	1	76.56%	96.78%	148.0 f	140.6 f	68.6 f

*0, mutated type allele; 1, wild-type allele. The mutated alleles of BnFLC.A2 and BnFLC.C2 and the wild-type allele of qFT.A3 (BnFLC.A3b) were all from R11. Letters on the right side of the numbers indicate the significance of multiple comparisons.*

### Allelic Variations in *BnFLC.A2, BnFLC.C2*, and *BnFLC.A3b* Had Additive Effects on FT as Well as in Seed Yield and Quality Trait in the Eight Genotypes

The FT of the eight genotypes was investigated in three environments, and significant differences were consistently observed in every condition (Figure 4 and Table 1). ID01-000, possessing all the three mutated alleles at *BnFLC.A2*, *BnFLC.C2*, and *BnFLC.A3b*, flowered earliest, whether in the winter environments (Jiangling and Wuhan) or in the spring environment (Minle) (Table 1), while ID08-111, carrying the WT genotypes at all loci, had the latest FT in all environments (Table 1). Genotypes ID02-010, ID03-011, and ID04-100, which possessed only one WT allele, flowered later than genotype ID01-000, but earlier than other genotypes (Table 1). Genotypes ID05-011, ID06-110, and ID07-101, which possessed two of the WT alleles, flowered earlier than genotype ID08-111, but later than other genotypes (Table 1). The broad-sense heritability of FT in the Jiangling, Wuhan, and Mingle reached 0.8605, indicating that the eight genotypes exhibited a stable genetic effect on FT.

Compared to 616R (i.e., ID06-110), the other seven genotypes also exhibited widely phenotypic alterations in multiple traits (Figure 4 and Supplementary Figure 3). Plant architecture traits, such as BN, BH, PH, and SH, showed a tendency from low to high with the delaying of FT (Supplementary Figure 3). Yield component traits, including MSN, PSN, SL, and SSN, were significantly lowered in early FT genotypes than in late FT genotypes (Supplementary Figure 3). Thus, ID01-000, with the earliest FT, had the lowest PY, while the latest FT ID08-111 had the highest PY, implying an obvious conflict between early FT and PY (Supplementary Figure 3). Similar changes were also found for OC, i.e., the genotypes showing earlier FT but sharing lower seed OC; in contrast, seed PC was raised with the postponed FT in the eight genotypes (Supplementary Figure 3). In addition, other traits, including ML, SL, and GC, also displayed a significant difference among the eight genotypes, though not exhibiting identical rules (Supplementary Figure 3).

**FIGURE 5:**
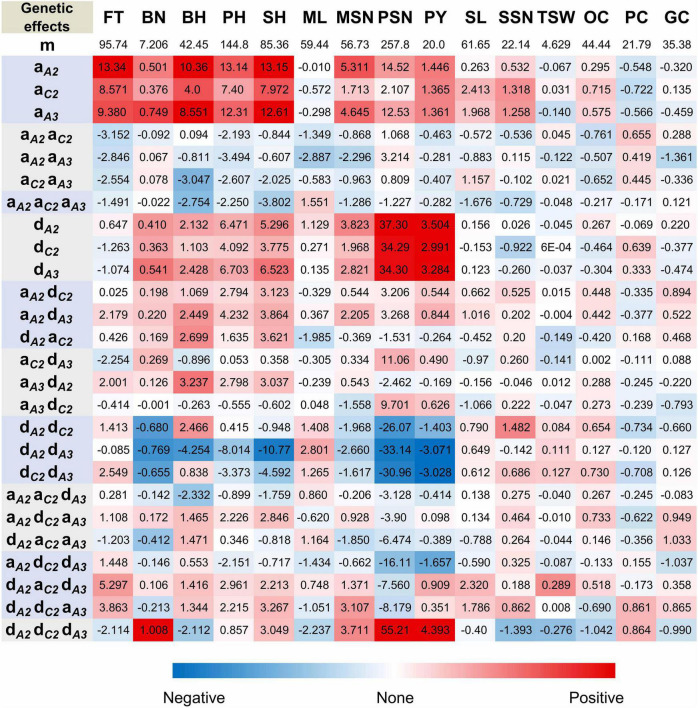
Genetic analysis of the effects of *BnFLC.A2*, *BnFLC.C2*, and *qFT.A3* (*BnFLC.A3b*). m, mean effect; a, additive effect; d, dominant effect; others, interaction effects; the footnotes *A2*, *BnFLC.A2*; *C2*, *BnFLC.C2*; A3, *qFT.A3* (*BnFLC.A3b*); FT, flowering time (days); BN, branch number; BH, branch height (cm); PH, plant height (cm); SH, stem height (cm); ML, main raceme length (cm); MSN, silique number on main raceme; PSN, silique number per plant; PY, plant yield (g); SL, silique length (mm); SSN, seed number per silique; TSW, thousand seed weight (g); OC, oil content (%); PC, protein content (%); GC, glucosinolate content (μmol/g). The red color indicates positive effects, while the blue color indicates negative effects. Best linear unbiased estimation values of the phenotypes for the 15 traits of the 27 NIL genotypes in the Jiangling, Wuhan, and Minle environments were used in the calculation.

Since the above observations clearly demonstrated that the three *BnFLC* homologs greatly contributed to FT variations in NIL backgrounds, the individual genetic effect of these homologs on FT and other traits were dissected by the additive-epistasis model *via* the BLUE values of the eight genotypes (Supplementary Table 3). The results showed that *BnFLC.A2*, *BnFLC.C2*, and *BnFLC.A3b* exerted the positive additive effects on FT, among which the additive effect of *BnFLC.A2* is greater than that of *BnFLC.C2* and *BnFLC.A3b* (Figure 5), suggesting that *BnFLC.A2* may be a functional *FLC* homolog with a stronger influence on FT than the other two in rapeseed. However, the additive × additive and additive × additive × additive effects between these three homologs were negative, indicating dose effects among the three genes (Figure 5).

Similar to FT, positive additive effects that were also detected on plant architecture traits BN, BH, PH, and SH, and *BnFLC.A2* showed higher additive effects than *BnFLC.C2* and *BnFLC.A3b* in these traits (Figure 5). For seed quality traits, positive additive effects were detected on OC and negative additive effects were detected on PC, and *BnFLC.C2* showed stronger effects on OC and PC than *BnFLC.A2* and *BnFLC.A3b* (Figure 5). As for GC, it was not affected by the additive effect of any of the three genes, which coincided with its null correlation with FT (Figure 5 and Table 2). For yield component traits, *BnFLC.A2* and *BnFLC.A3b* exerted higher positive additive effects on MSN and PSN than *BnFLC.C2*, coincided with the less silique number phenotype of ID02-010 (Figure 5 and Supplementary Table 3), while *BnFLC.C2* and *BnFLC.A3b* exerted greater positive additive effects on SL and SSN than *BnFLC.A2*, which corresponded with the longer SL and higher SSN of genotype ID05-011 (Figure 5 and Supplementary Table 3). In addition, *BnFLC.A2* and *BnFLC.A3b* exerted negative additive effects on TSW (Figure 5). At last, the additive effects of *BnFLC.A2*, *BnFLC.C2*, and *BnFLC.A3b* on PY were 1.446, 1.365, and 1.361 g, respectively, coincided with the significant Pearson correlation coefficients of FT and PY (Table 2), which indicated the acceleration of FT was associated with a severe yield loss, while the delay of FT was associated with yield gain (Figure 5).

**TABLE 2:** The Pearson correlation coefficients between flowering time and other traits.

FT	BN	BH	PH	SH	ML	MSN	PSN	PY	SL	SSN	TSW	OC	PC	GC
flowering time	branch number	branch height	plant height	stem height	main raceme length	silique number on main raceme	silique number per plant	plant yield	silique length	seed number per silique	thousand seed weight	oil content	protein content	glucosinolate content
Pearson correlation coefficients (the eight genotypes)	0.89**	0.95**	0.99**	0.97**	0.055	0.95**	0.84**	0.98**	0.63	0.82*	−0.42	0.74*	−0.85**	−0.13
Pearson correlation coefficients (the 19 NIL hybrids)	0.93**	0.98**	0.99**	0.98**	−0.32	0.96**	0.78**	0.89**	0.86**	0.86**	−0.70**	0.89**	−0.91**	−0.017
Pearson correlation coefficients (the 27 NIL genotypes)	0.9**	0.97**	0.97**	0.97**	−0.16	0.93**	0.58**	0.76**	0.74**	0.84**	−0.59**	0.82**	−0.88**	−0.062
Pearson correlation coefficients (the 16 non-NIL hybrids)	0.55*	0.96**	0.87**	0.93**	−032	0.51*	0.42	0.72**	0.25	0.64**	0.12	0.20	−0.46	0.082

*significant at p < 0.05; **significant at p < 0.01.

### Heterozygous Status in *BnFLC.A2, BnFLC.C2, and BnFLC.A3b* Generated Accumulative Heterosis in Plant Yield *via* Nonadditive Effects

As described above, there is an obvious conflict between FT and PY in homozygous NIL backgrounds. To test whether this conflict remained in their hybrids, we mutually crossed the eight genotypes and constructed a total of 19 NIL hybrids with heterozygous genotypes at least at one locus of *BnFLC.A2*, *BnFLC.C2*, and *BnFLC.A3b*, corresponding to genotypes from ID09-020 to ID027-121 (Supplementary Table 4). We investigated the FT and PY of these hybrids and performed multiple comparisons of both traits. Abundant variations in FT and PY were retained in different hybrids, ranging from 70.40 to 115.1 days, and from 18.83 to 26.78 g, respectively (Supplementary Figure 4). Compared to the original eight homozygous genotypes, the difference of FT in hybrid NILs was narrowed down, whereas that of PY was amplified and predisposed to the high-value end (Supplementary Figure 4). Consistently, the Pearson correlation coefficient between the PY and FT reduced to 0.76 when the 19 NIL hybrids joined into the analysis, rather smaller than that 0.98 in the eight homozygous genotypes (Table 2).

**FIGURE 6:**
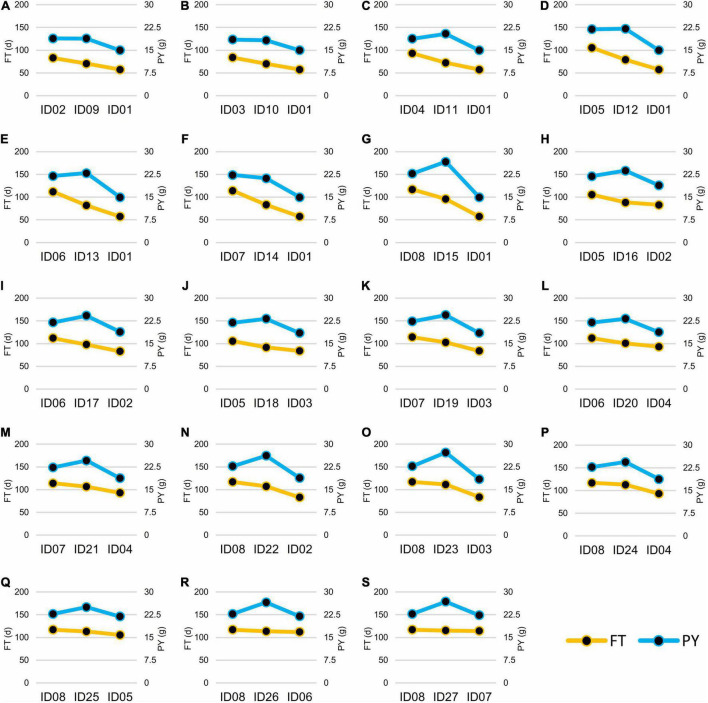
Flowering time (FT) and plant yield (PY) of the 19 NIL hybrids and their crossing parents. For each of the box from **(A–S)**, the left side was the female parent, the right side was the male parent, and the middle was the hybrid. The left vertical axis and the yellow line showed FT (d), while the right vertical axis and the blue line showed PY (g).

According to the FT phenotypes, the hybrids can be classified into two groups. In Group I, the FT of hybrids was lower or had no significant difference than the mid-parent value. In Group II, the FT of hybrids was significantly higher than the mid-parent value (Figure 6). In Group I, 12 of the 16 hybrids showed no significance of PY to the high-value parents of PY, except for ID16-012, IDh18-021, IDh26-112, and IDh-121, whose PY was significantly higher than their high-value parents (Figure 6). In Group II, 6 of the 7 hybrids showed significantly higher PY than the high-value parents, except for ID24-122, whose PY showed no significance to its high-value parent (Figure 6). In addition, we noticed that the mid-parent heterosis for plant architecture traits and yield component traits was also observed in these hybrids (Table 3). Among them, PSN and PY showed stronger heterosis performances than the other traits (Table 3), suggesting the heterosis of PSN was the most important component of the heterosis of PY. Finally, the highest heterosis was found in the triple heterozygous genotype ID15-222 from group II, reaching 25.05 and 41.49% for PSN and PY, respectively (Table 3), indicating the importance of the increasing of the heterozygosity for the heterosis of PSN and PY. These findings suggested that in the NIL background, FT was largely inherited as an additive manner, while the PY was consistently inherited as a dominant manner. As a result, the conflict between early FT and high PY can be greatly overcome by the heterosis derived from *BnFLC.A2*, *BnFLC.C2*, and *BnFLC.A3b* in the hybrid-NIL backgrounds.

**TABLE 3:** Analysis of mid-parent heterosis of the 19 NIL hybrids.

Mid-parent heterosis of the hybrids	FT Flowering time	BN Branch number	BH Branch height	PH Plant height	SH Stem height	ML Main raceme length	MSN Silique number on main raceme	PSN Silique number per plant	PY Plant yield	SL Silique length	SSN Seed number per silique	TSW Thousand seed weight	OC Oil content	PC Protein content	GC Glycosylate content
ID09-020	0.33%	5.61%	7.76%	3.52%	6.95%	−0.12%	8.79%	7.47%	11.35%	0.66%	−5.89%	0.48%	−1.05%	2.53%	1.35%
ID10-002	−1.02%	−1.45%	−5.17%	1.25%	0.86%	1.59%	0.15%	6.95%	9.19%	0.37%	−2.26%	1.45%	−1.13%	2.43%	−3.25%
ID11-200	−3.97%	−4.83%	−8.69%	1.95%	−3.47%	7.56%	3.64%	14.27%	21.03%	−0.04%	0.70%	1.02%	1.28%	−1.47%	2.84%
ID12-022	−2.92%	−1.80%	1.86%	2.21%	−3.16%	9.07%	1.47%	18.44%	19.76%	−5.20%	−9.78%	2.07%	−0.83%	0.89%	−1.84%
ID13-220	−3.53%	3.92%	−3.98%	4.87%	−0.71%	12.23%	2.50%	17.84%	24.19%	−1.44%	−3.06%	-0.53%	2.61%	−5.89%	−2.32%
ID14-202	−3.06%	−6.52%	−12.02%	1.29%	−10.34%	18.08%	6.77%	12.32%	13.85%	−1.27%	−8.62%	2.32%	0.64%	−1.42%	1.31%
ID15-222	9.89%	2.27%	16.45%	11.31%	7.09%	17.56%	19.40%	25.05%	41.49%	3.38%	0.00%	0.48%	4.44%	−5.64%	−1.82%
ID16-012	−6.15%	10.22%	3.93%	2.42%	5.89%	−2.32%	2.14%	18.51%	16.41%	−3.11%	−2.03%	−2.85%	−2.22%	4.02%	−2.32%
ID17-210	0.28%	12.80%	0.30%	3.48%	8.09%	−2.98%	8.70%	18.13%	18.61%	1.06%	0.03%	−3.39%	−1.30%	3.31%	−0.34%
ID18-021	−2.97%	−0.11%	−4.09%	−1.01%	−3.27%	2.04%	−1.82%	17.69%	14.73%	−3.14%	−7.42%	−1.10%	−3.04%	6.35%	−8.23%
ID19-201	3.46%	10.39%	2.40%	4.78%	6.01%	2.84%	10.86%	16.02%	19.55%	2.07%	−2.18%	3.62%	1.85%	−0.58%	−4.27%
ID20-120	−1.88%	5.66%	2.15%	3.50%	5.38%	0.82%	5.26%	12.35%	13.80%	2.37%	−5.08%	1.51%	−2.29%	5.45%	0.98%
ID21-102	2.97%	8.55%	16.64%	7.71%	12.90%	−0.08%	8.00%	11.01%	19.73%	3.28%	−2.71%	3.11%	-0.29%	1.46%	0.12%
ID22-212	7.30%	8.93%	16.54%	10.62%	13.10%	6.71%	16.23%	14.68%	26.03%	7.13%	3.87%	4.45%	2.06%	−1.04%	5.91%
ID23-221	10.84%	1.51%	26.23%	13.02%	19.79%	2.30%	13.63%	16.56%	32.24%	5.88%	13.05%	0.35%	2.97%	−1.25%	−3.89%
ID24-122	7.53%	5.99%	30.29%	11.21%	19.21%	−1.15%	12.55%	10.45%	17.66%	3.57%	6.35%	0.92%	3.62%	−2.64%	−0.37%
ID25-211	1.68%	3.47%	18.36%	6.95%	10.72%	0.12%	3.46%	9.82%	12.02%	−1.85%	1.80%	−4.97%	0.62%	−2.40%	4.33%
ID26-112	−0.76%	11.11%	2.90%	6.18%	8.51%	1.84%	8.20%	16.52%	18.81%	0.48%	2.03%	−4.77%	0.91%	−1.89%	0.15%
ID27-121	−0.47%	8.58%	5.57%	5.13%	8.27%	−1.12%	2.92%	15.03%	18.91%	−0.67%	1.20%	−0.97%	2.21%	−2.65%	2.02%

To support the above conclusion, we further dissected the genetic effects of *BnFLC.A2*, *BnFLC.C2*, and *BnFLC.A3b* on FT, PY, and PY component traits by the BLUE values of the 27 NIL genotypes using the additive–dominant–epistasis model (Supplementary Table 3). The results showed that there are very weak nonadditive effects ranging from –2.254 to 5.297 days on FT existed for the three *FLC* homologs (Figure 5), indicating less mid-parent heterosis for FT. In contrast, *BnFLC.A2*, *BnFLC.C2*, and *BnFLC.A3b* had much stronger dominant effects on PY (3.504, 2.991, and 3.284 g, respectively) than their additive effects (1.446, 1.365, and 1.361 g, respectively; Figure 5), indicating the dominant effects were the important reason for PY heterosis (Figure 5 and Table 3). We also noticed that the dominant × dominant × dominant effect of *BnFLC.A2*, *BnFLC.C2*, and *BnFLC.A3b* on PY was 4.393 g, a huge interaction for the gain of PY, which was coincided with the highest mid-parent heterosis and the highest PY of ID15-222. Therefore, we confirmed that the genotype which was heterozygous on *BnFLC.A2*, *BnFLC.C2*, and *BnFLC.A3b* could simultaneously be the most preponderant genotype in the 27 NIL genotypes for PY heterosis.

### Heterosis in Plant Yield Is Also Associated With Heterozygous *BnFLC.A2, BnFLC.C2*, and/or *BnFLC.A3b* Genotypes in Non-NIL Hybrids

**FIGURE 7:**
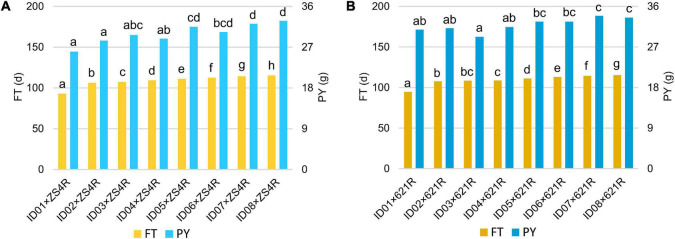
Multiple comparisons of FT and PY of the non-NIL hybrids. **(A)** Multiple comparisons of the hybrids produced by crossing with ZS4R. **(B)** Multiple comparisons of the hybrids produced by crossing with 621R. The left bars indicate FT (d), and the right bars indicate PY (g). The left legend stands for FT (d), and the right legend stands for PY (g), and the lower-case letters next to the bars showed the significance of multiple comparisons.

To test whether the heterosis of PY mediated by *BnFLC.A2*, *BnFLC.C2*, and *BnFLC.A3b* can be explored in early-flowering hybrid breeding, we investigated the FT and PY of 16 non-NIL hybrids which were constructed by crossing two commercial pol CMS restorers (i.e., ZS4R and 621R) to the eight genotypes (i.e., ID01–ID08). Genotyping by PCR analysis showed that ZS4R carries the same alleles in the *BnFLC.A2*, *BnFLC.C2*, and *BnFLC.A3b* loci as 616A (*Bnflc.a2/Bnflc.c2/BnFLC.A3b*), thus designated as 110, while 621R has the late-flowering alleles in all the three genes (*BnFLC.A2/BnFLC.C2/BnFLC.A3b*), thus designated as 111. As shown in Figure 7, we observed that in both hybrid groups, the resulting hybrids exhibited significant differences in FT and displayed a similar tendency as the eight genotypes (Figure 4 and Supplementary Figure 3), varying from 93.05 to 115.5 days for the ZS4R hybrid group and from 9.65 to 115.6 days for the 621R hybrid group, respectively. Accordingly, these results indicate that the different alleles and their permutations also have a significant and identical effect on FT in non-NIL hybrids. Consistently, it can easily be noticed that with the extension of FT, the PY performance was also gradually increased in the non-NIL hybrids (Figures 7A,B), with a significant Pearson correlation coefficient of 0.72 (Table 2), showing that early flowering caused yield loss in non-NIL hybrids as well.

However, we also observed that the heterozygous status of the *BnFLC* homologs successfully made up the yield reduction in some hybrids. For example, ID06 × ZS4R-110 from the ZS4R hybrid group, homozygous in all the *BnFLC.A2*, *BnFLC.C2*, and *BnFLC.A3b* genes, had an FT of 112.5 days, which was significantly later than ID02 × ZS4R-210, ID03 × ZS4R-222, ID04 × ZS4R-120, and ID05 × ZS4R-212, while there was no significant difference in PY between ID06 × ZS4R-110 and the other four hybrids (Figure 7A). Consistently, it was also clearly revealed that ID01 × 621R-222 from the 621R hybrid group, which is heterozygous in all the *BnFLC.A2*, *BnFLC.C2*, and *BnFLC.A3b* genes, showed the earliest FT phenotype among all the hybrids in the same group, while ID01 × 621R-222 displayed the same PY level as ID02 × 621R-212, ID03 × 621R-221, ID04 × 621R-122, ID05 × 621R-211, and ID06 × 621R-112, suggesting that a 12–17-day FT difference did not cause obvious yield loss in these hybrids (Figure 7B). Similarly, ID05 × 621R-211, ID06 × 621R-112, and ID07 × 621R-121 had no significant difference in PY with ID08 × 621R-111, which is homozygous in all the *BnFLC.A2*, *BnFLC.C2*, and *BnFLC.A3b* genes, but their FT was 1.2–4.4 days earlier than that of ID08 × 621R-111, respectively (Figure 7B). In summary, all these results showed that the exploration of the heterosis of *BnFLC* homologs in complex genetic background can effectively rescue the PY damage during early-flowering breeding as well.

## Discussion

In this study, we discovered *qFT.A3* as an early flowering factor of pol CMS line 616A by using a QTL mapping strategy. According to the latest genome information of ZS11 (Song et al., 2020a), *BnFLC.A3b* is the only annotated FT gene in the 727 kb candidate region of *qFT.A3*. Similarly, *BnFLC.A3b* was previously colocalized with an FT QTL located on chromosome A3 from a DH population that derived the F_1_ cross of Ningyou7 and Tapidor (Wang et al., 2011; Zou et al., 2012). *BnFLC.A3b* is expressed normally in the winter-type varieties, such as Darmor, Tapidor, and Quinta, but is considerably less expressed in the spring- or semi-winter-type varieties, such as Westar, ZS11, and Ningyou7 (Zou et al., 2012; Song et al., 2020b; Yin et al., 2020). Alternative splicing induced by intron variation was probably responsible for the differential expression of *BnFLC.A3b* between Ningyou7 and Tapidor, hinting that *BnFLC.A3b* was thus presumed to be a functional FT gene (Zou et al., 2012). Here, we also identified rich InDel and SNP variations in *BnFLC.A3b* between R11 and 616A, and observed a 13-fold higher expression level of *BnFLC.A3b* in the former parents than the latter. Therefore, even with the mutant alleles *Bnflc.a2* and *Bnflc.c2*, which greatly promote early flowering, R11 displayed a significant late-flowering phenotype than 616A in the winter environment. All these suggest that *BnFLC.A3b* is the most likely target gene of *qFT.A3*, which acts as a key FT promoting factor even under the existence of functional *BnFLC.A2* and *BnFLC.C2.*

The rational design of FT enables breeders to adapt varieties to various environments. As a subtropical region, central China is suitable for semi-winter rapeseed, indica rice, and maize cultivation. “Rice—ratoon rice—rapeseed,” “double season rice—rapeseed,” and “maize—rapeseed” farming systems are being promoted in this region to simultaneously produce rice, corn, and rapeseed. However, the limitations of growth season require rapeseed to be sowed later and harvested earlier for saving time for rice or corn, because rice and corn are more important in China (Xu et al., 2018; Zhao and Yang, 2018; Yuan et al., 2019). As a warm temperate region, northwestern China is suitable for one season spring rapeseed, in which rapeseed needs to be harvested earlier for escaping the early frost. In this study, we employed natural variants of *BnFLC.A2*, *BnFLC.C2*, and *BnFLC.A3b* to create sterile lines with the 616A background in an effort to adapt rapeseed hybrids to different farming systems and environments.

As flowering is the critical transition from vegetative to reproductive growth, besides FT, FT genes can affect many other agronomic traits. Among the traits affected in this study, the FT genes did not only have additive effects on PY and plant architecture component traits but also had dominant effects on these traits. Smaller plant architecture corresponds to lower dry matter production, indicating that a correlation between early FT and yield loss may be an indirect reflection of plant architecture and dry matter production (Miedaner et al., 2018). Suggesting the yield loss induced by early flowering was a comprehensive reflection in the improvement of FT.

The heterosis or nonadditive effects of FT genes afforded us an opportunity to balance yield when FT was advanced, but this also complicated the influence of FT on other agronomic traits. In this study, the nonadditive effects of *BnFLC.A2*, *BnFLC.C2*, and *BnFLC.A3b* successfully increased heterosis, providing an additional example of FT gene heterosis as well as an innovative germplasm for overcoming the conflict between early flowering and yield loss in rapeseed breeding. In previous studies, heterotic QTLs *Ghd7* and *Ehd3* resulted from the introgression of subpopulation alleles and diversity selection in rice (Lin et al., 2020). Yield heterosis in tomatoes resulting from heterozygous mutations of *SFT* (*sft*/*+*) was hypothesized to be based on altered amounts of functional SFT proteins within each modular sympodial unit (Krieger et al., 2010; Park et al., 2014). Response of genes to carbohydrate stimulus and nutrient levels might be involved in the generation of the large plant phenotype of F_1_ mimics in *Arabidopsis*. In the current study, the heterosis of the *BnFLC* homologs might be embodied through downstream *FT* paralogs, similar as in tomatoes. We will pursue research investigating the mechanism of FT gene heterosis in rapeseed in the future.

In this study, the elite line 616A is the female parent of several registered cultivars, including Shengguang136 (616A crossed with ZS4R) and Shengguang 128 (616A crossed with 621R). Shengguang 136 is suitable for the climate of central China, and Shengguang 128 is adaptable to both environments of central China and northwestern China, but more maturity types are still required. In the Jiangling and Wuhan environments representing the climate in central China, IDh61-222 (ID01-000 × 621R) was shown to be appropriate for commercial use because of its suitable early flowering and harvest times. In the Minle environment, which represents the climate in northwestern China, IDh67-121 (ID07-101 × 621R) and IDh68-111 (ID06-110 × 621R) were suggested to gain a higher yield without wasting light and temperature conditions. In the future, the eight genotypes of male sterile lines of 616A will be crossed with additional restore lines and tested in more environments to breed hybrids adaptable to different farming systems in different sub-environments (Supplementary Figure 5).

## Conclusion

In this study, we dissected the genetics of the early FT of the elite lines 616A and R11, and classified that yield loss induced by early flowering could be compensated by the heterosis of FT genes *BnFLC.A2*, *BnFLC.C2*, and *BnFLC.A3b*. These results not only indicated that the dominant effect is an important contributor to heterosis but also supported a feasible method to facilitate yield *via* heterosis of FT genes during the rational design of elite cultivars suitable for the current farming systems and different sub-environments. Moreover, the results suggested that the sterile lines of the eight genotypes developed in the current study could be potential for hybrid breeding in the near future.

## Data Availability Statement

The datasets presented in this study can be found in online repositories. The sequences of BnFLC.A3b-616A and BnFLC.A3b-R11 were deposited at GenBank (https://www.ncbi.nlm.nih.gov/) with the accession numbers of OL674083 and OL674084, respectively.

## Author Contributions

CF, GY, DH, and FD conceived and designed the experiments. CF, ZW, YS, and AA performed the experiments. CF and PW analyzed the data. CF, GY, and DH wrote this manuscript. All authors contributed to the article and approved the submitted version.

## Conflict of Interest

The authors declare that the research was conducted in the absence of any commercial or financial relationships that could be construed as a potential conflict of interest.

## Publisher’s Note

All claims expressed in this article are solely those of the authors and do not necessarily represent those of their affiliated organizations, or those of the publisher, the editors and the reviewers. Any product that may be evaluated in this article, or claim that may be made by its manufacturer, is not guaranteed or endorsed by the publisher.

## Footnotes

1
http://broadinstitute.github.io/picard/
