# Supplementary material for: Heterosis Derived From Nonadditive Effects of the BnFLC Homologs Coordinates Early Flowering and High Yield in Rapeseed (Brassica napus L.)
Source: Front Plant Sci. 2022 Feb 15;12:798371. doi: 10.3389/fpls.2021.798371 (PMC8893081; doi:10.3389/fpls.2021.798371)
Supplement: Supplementary file 1 [file Data_Sheet_1.pdf]

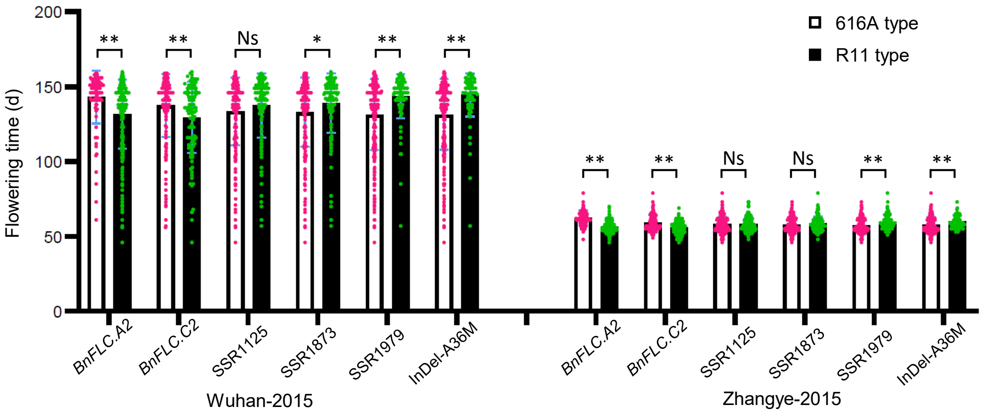

Supplementary Figure 1. Single marker analysis of the inner-gene markers and linkage markers in the DH population built by 616A and R11 in Wuhan-2015 and Zhangye-2015 environments, respectively.

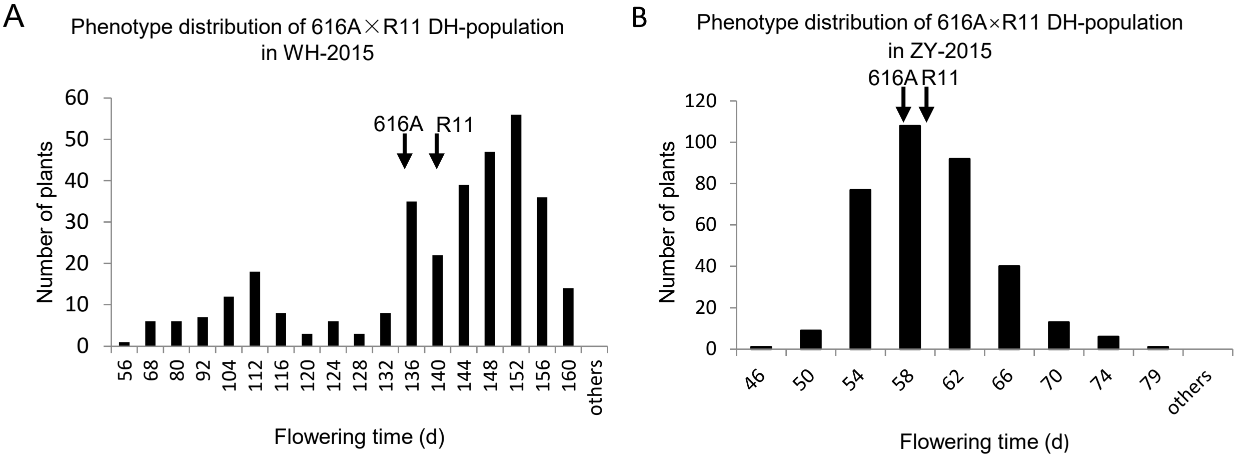

Supplementary Figure 2. Histogram of phenotype distribution of the DH population constructed by crossing 616A and R11 in the Wuhan-2015 and Zhangye-2015 environments.

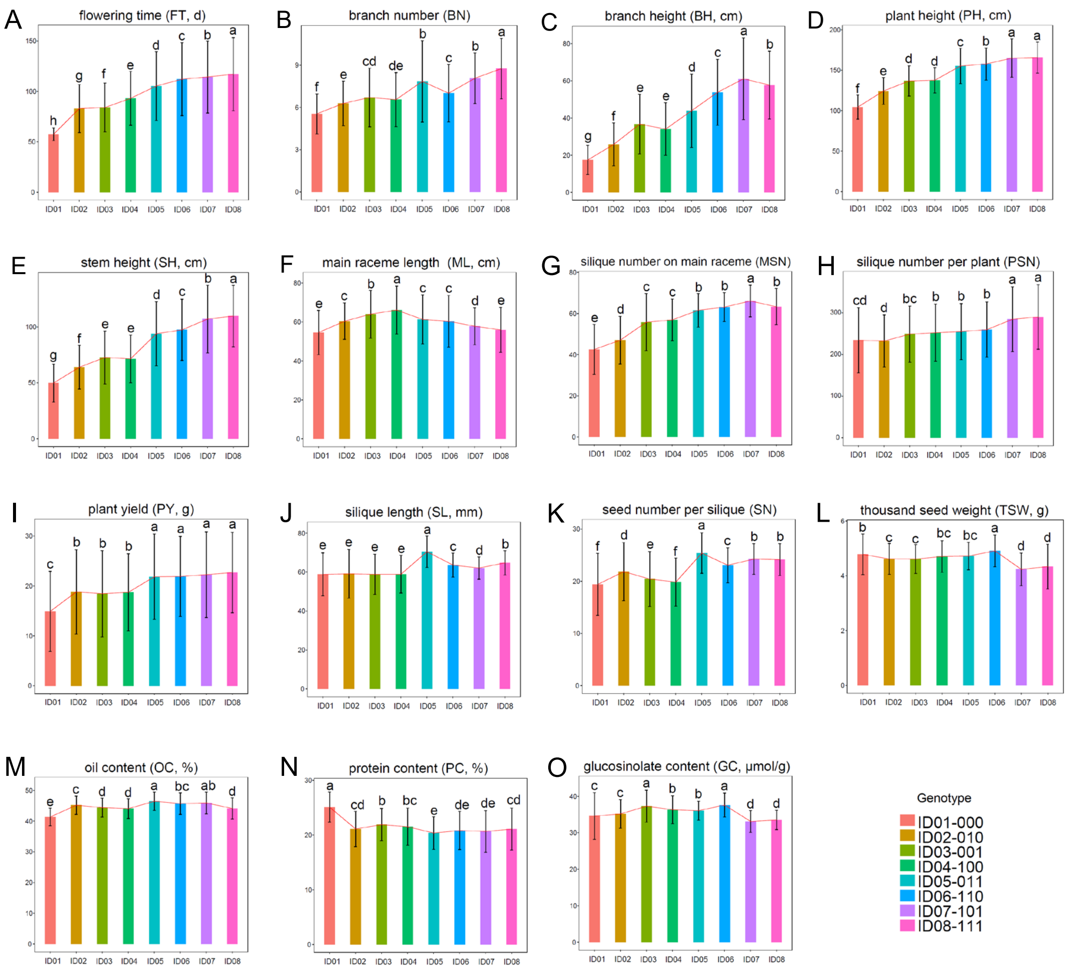

Supplementary Figure 3. Multiple comparisons of multiple traits of the 8 genotypes. The same lower case indicates no significance. The red line was the tendency line.

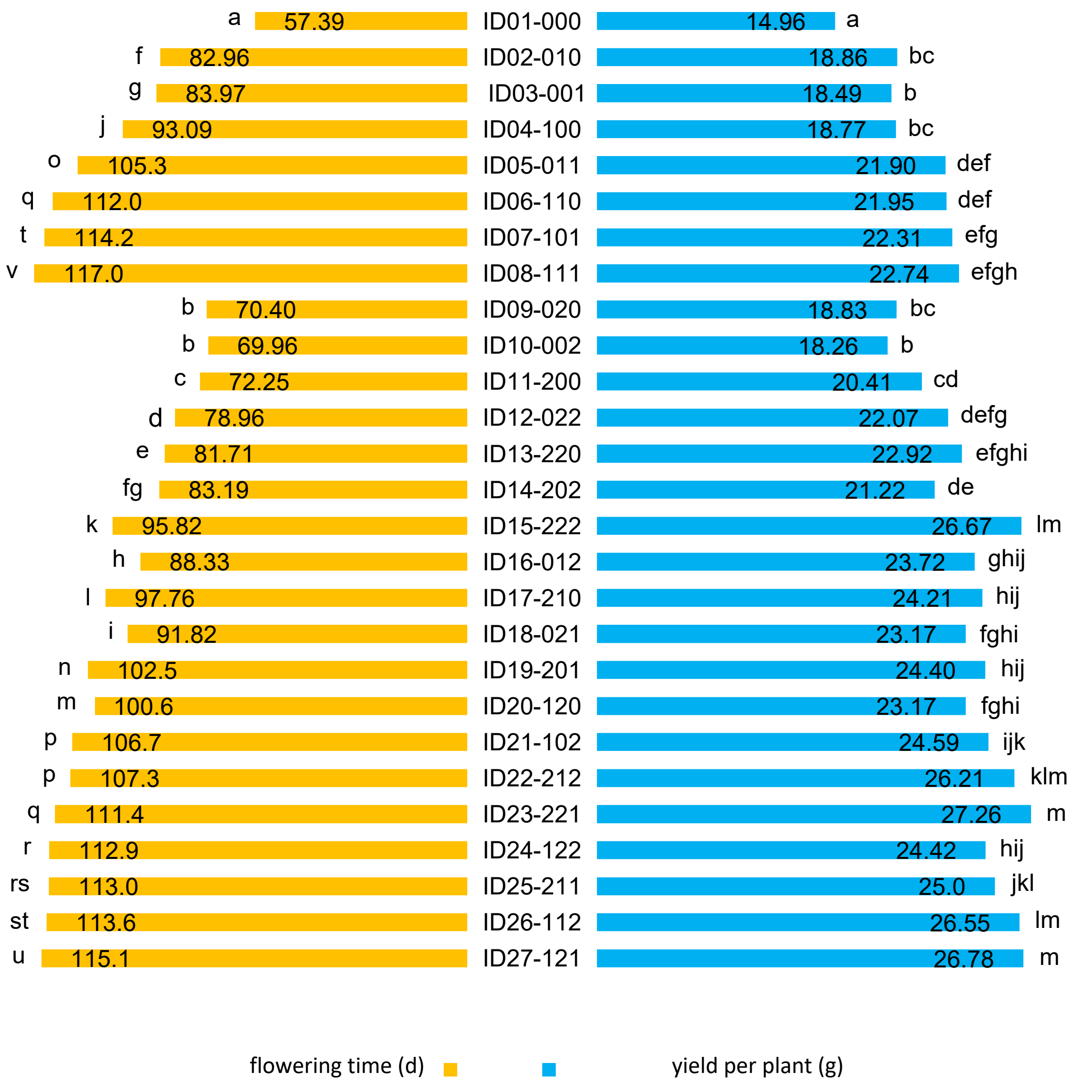

Supplementary Figure 4. Symmetric bar statistics graph of the twenty-seven genotypes. The left legend stands for flowering time (FT, d), and the right legend stands for yield per plant (PY, g). The lower-case letters next to the bars showed the significance of multiple comparisons.

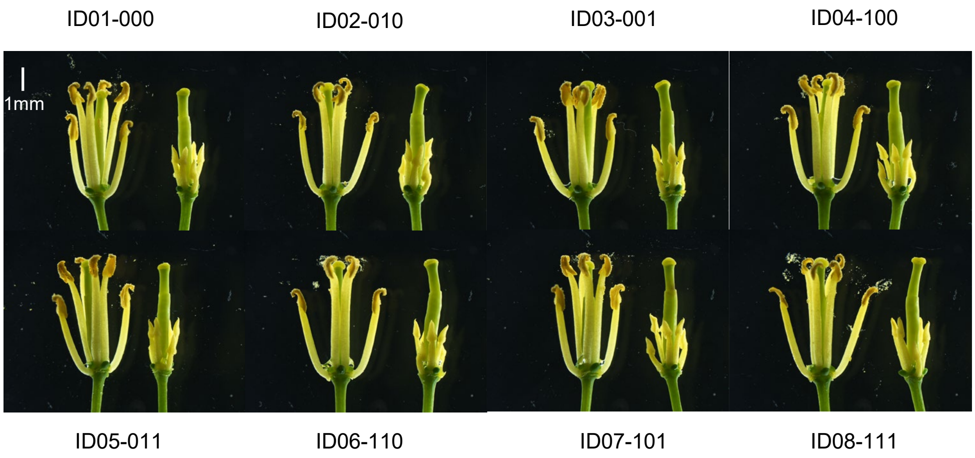

Supplementary Figure 5. Floral organ morphology of the near isogenic lines of the eight genotypes (left image of each pair) and their corresponding sterile lines (right image of each pair) in the Wuhan environment, March 12, 2021.
